# Dengue Types 1 and 3 Identified in Travelers Returning from Kathmandu, Nepal, during the October 2022 Outbreak Are Related to Strains Recently Identified in India

**DOI:** 10.3390/v15122334

**Published:** 2023-11-28

**Authors:** Neta S. Zuckerman, Eli Schwartz, Prativa Pandey, Oran Erster, Osnat Halpern, Efrat Bucris, Hagar Morad-Eliyahu, Marina Wax, Yaniv Lustig

**Affiliations:** 1 Central Virology Laboratory, Ministry of Health, Chaim Sheba Medical Center, Tel Hashomer 52621, Israel; oran.erster@sheba.health.gov.il (O.E.); osnat.halpern@sheba.health.gov.il (O.H.); efrat.bucris@sheba.health.gov.il (E.B.); hagar.morad@sheba.health.gov.il (H.M.-E.); marina.wax@sheba.health.gov.il (M.W.); yaniv.lustig@sheba.health.gov.il (Y.L.); 2 The Center for Travel and Tropical Medicine, Sheba Medical Center, Tel Hashomer 52621, Israel; eli.schwartz@sheba.health.gov.il; 3 Sackler School of Medicine, Tel Aviv University, Tel Aviv 39040, Israel; 4 CIWEC Hospital and Travel Medicine Center, Kathmandu 44600, Nepal; prativapandey@ciwec-clinic.com

**Keywords:** dengue, outbreak, phylogenetic analysis, travel, next generation sequencing

## Abstract

Phylogenetic analysis of dengue serotypes 1 and 3, which were diagnosed in travelers and Nepalese infected in Kathmandu during the October 2022 outbreak, revealed that both serotypes were clustered closest to the sequences sampled in India. This suggests both serotypes may have originated in India.

## 1. Introduction

Dengue virus (DENV) is a vector-borne virus belonging to the flavivirus genus of the *Flaviviridae* family, transmitted by *Aedes aegypti* or *Aedes albopictus* mosquitos [1]. Dengue infections result in a spectrum of clinical manifestations, ranging from asymptomatic or mild illness to severe and potentially life-threatening conditions. Symptoms characterizing dengue fever include non-specific flu-like symptoms, such as chills, muscle pain, appetite loss, lethargy, enlarged lymph nodes, headaches, fever, and a skin rash [2]. Presently, there are no widely distributed antiviral treatments or vaccines, though progress is being made in the research and development of new antivirals and antiviral molecules targeting DENVs [3,4]. Thus, approaches to managing dengue infections include providing symptomatic treatments and implementing programs for vector control [5]. DENV is classified into four distinct serotypes: DENV-1, DENV-2, DENV-3, and DENV-4, each possessing unique antigenic characteristics. Although the four serotypes share a common genomic structure and the same range of clinical symptoms, each serotype possesses a unique antigenic profile, making cross-protection limited and allowing for reinfection with a different serotype. The presence of multiple serotypes is a key factor in the complexity of dengue epidemiology, as infection by different DENV serotypes increases the risk of developing severe dengue and dengue hemorrhagic fever due to a phenomenon called antibody-dependent enhancement [2,6]. The prevalence of each serotype varies within different regions and changes over time due to population immunity, mosquito abundance, and viral introductions from other areas [1].

DENV has been responsible for a growing share of vector-borne diseases, leading to an estimated 400 million infections annually, and has become a major public health concern in many tropical and subtropical regions of the world [7,8]. Indeed, the transmission of DENV generally occurs at lower altitudes in tropical and subtropical regions, as the *Aedes* mosquitoes transmitting the disease have been generally known to thrive in lowland areas, where the high temperatures, climate, and environmental conditions favor their survival and growth [9]. However, there is a noticeable trend in *Aedes* mosquito populations that have established themselves in regions previously unaffected by dengue [10]. Indeed, in recent years, dengue transmission has been reported to expand into areas located at higher altitudes, significantly widening the geographic reach of the virus and rendering DENV endemic in the majority of Southeast Asia, and Central and South America. These geographic shifts in DENV transmission raise significant concerns and implications within the field of public health as they highlight the need to understand the dynamics of this disease in new ecological contexts. In addition, over the past decade, there has been a notable increase in autochthonous transmission of DENV within Europe, escalating from a single case in France in 2010 to at least 60 cases by 2022 [8,11].

The DENV vectors are the *Aedes* mosquitoes, mainly *Aedes egypti*, but also *Aedes albopictus* to a lesser extent. In fact, *Aedes* albopictus, which was initially identified in France in 2004 [12], extended its presence northward and is now found throughout Europe. Indeed, one of the first autochthonous transmissions was reported in France in 2010 [13] and many additional autochthonous outbreaks have been reported throughout Europe in recent years [14], including in France, Italy, and Spain. The environmental conditions conducive to their accelerated development are becoming more widespread due to elevated temperatures resulting from climate change. This temperature rise contributes to an enhanced survival rate of *Aedes* mosquitoes, an increased frequency of biting, and a more favorable environment for the development of the virus. Together with the large number of DENV-carrying travelers returning from dengue-endemic countries, dengue is increasingly seen as a public health threat in Europe as well [11,13]

The first case of DENV in Nepal was identified in a traveler in 2004 [15]. Dengue has established endemicity in Nepal, with all four serotypes in circulation and the identification of both DENV mosquito vectors in the country—the primary vectors *Aedes aegypti* and *Aedes albopictus* [16]. Notably, DENV serotypes 1 and 2 have emerged as the predominant contributors to the national disease burden, marked by significant outbreaks in 2010, 2013, 2016, and 2018 [17]. These outbreaks were predominantly reported in the low-lying regions of Nepal, adjoining India. However, in 2019, an unusually large DENV outbreak affected most of Nepal’s districts, including the first infection at higher altitudes, including Kathmandu, which is located approximately 1400 m above sea level. The 2019 epidemic was associated with serotypes 2 and 3 [17,18]. During that time, twelve Israeli travelers returning from Kathmandu were diagnosed with DENV2 and 3 in Israel. Partial sequencing of an isolate from one of the travelers revealed a close resemblance to DENV3 from a 2017 outbreak in Maharashtra, India [19]. In 2022, Nepal experienced its largest dengue virus (DENV) outbreak, with 54,784 cases and 88 deaths, affecting all districts of the country, including Kathmandu Valley, where the highest rate of infection was recorded [20]. This dengue outbreak represents the most extensively recorded occurrence in the recent history of small mountainous regions [21]. Kathmandu, located at a high altitude, was spared from mosquito-borne disease outbreaks; however, *Aedes* mosquitoes were recently established there, probably due to global climate change [22]. In October 2022, over 40 travelers in Nepal or returning from Nepal, all infected in Kathmandu, were diagnosed with DENV1 (74%) and DENV3 (25%) [23]. In this study, we sequenced the complete genomes of DENV from these patients’ samples to estimate the source of the importation of DENV serotypes into Kathmandu.

## 2. Materials and Methods

### 2.1. Study Patients

Forty-one non-Nepalese patients (travelers and expatriates) and twenty-eight local Nepalese were tested for DENV at CIWEC Hospital, Kathmandu, and Sheba Medical Center (SMC) in Israel. 

Ethical clearance was obtained from the Nepal Research Health Council (NHRC) and SMC IRB 9873-22-SMC. As data were assessed anonymously, informed consent was waived.

### 2.2. DENV Detection

Nucleic acids were tested after extraction from serum (among patients seen in Israel) or from the dried blood spots which were applied on Whatman filter paper. Extraction of nucleic acids from filter paper was performed by adding 700 µL of lysis buffer (MagNA Pure LC Total Nucleic Acid Isolation Kit lysis/binding buffer, Roche Diagnostics, Germany) to the dried blood spot in a 1.5 mL Eppendorf tube, followed by shaking at 400 rpm in room temperature for 30 min. Total nucleic acid content was extracted using magLEAD 12gC (Precision System Science Co., Ltd., Matsudo Chiba, Japan) in a 50 µL elusion buffer. DENV RNA was detected using a multiplex qRT-PCR of DENV1–4, as previously described [24]. Briefly, a multiplex RT-PCR reaction was carried out using 5 µL of RNA and the following primers and probes (each probe was distinctively fluorophore-labeled): Deng-1-F: CAAAAGGAAGTCGYGCAATA, Deng-1-R: CTGAGTGAATTCTCTCTGCTRAAC, Deng-2-F: CAGGCTATGGCACYGTCACGAT, Deng-2-R: CCATYTGCAGCARCACCATCTC, Deng-3-F: GGACTRGACACACGCACCCA, Deng-3-R: CATGTCTCTACCTTCTCGACTTGYCT, Deng-4-F: TTGTCCTAATGATGCTRGTCG, Deng-4-R: TCCACCYGAGACTCCTTCCA, Deng-1-P: CATGTGGYTGGGAGCRCGC, Deng-2-P: CTCYCCRAGAACGGGCCTCGACTTCAA, Deng-3-P: ACCTGGATGTCGGCTGAAGGAGCTTG, and Deng-4-P: TYCCTACYCCTACGCATCGCATTCCG. The cycling method and fluorescence capture were used to detect the FAM, HEX, Texas Red, and CY5 emissions in each well. The following thermal cycling parameters were used: reverse transcription (RT) at 50 °C for 30 min, RT inactivation at 95 °C for 2 min, fluorescence detection for 45 cycles at 95 °C for 15 s, and annealing at 60 °C for 1 min. 

### 2.3. Sequencing and Bioinformatics Analysis

Whole genomes of DENV were sequenced as follows. Libraries were prepared from nucleic acid extractions using the SMARTer stranded RNA-Seq kit (Takara Bio, Kusatsu, Shiga, Japan) and sequencing was carried out on the NovaSeq platform (Illumina, CA, USA). The resulting fastq files underwent quality control using FastQC and MultiQC [11], and low-quality sequences were filtered using trimmomatic [25]. Sequences were mapped to DENV1 (NC_001477.1) or DENV3 (NC_001475.2) reference genomes using BWA mem [26]. The resulting BAM files were sorted, indexed, and subjected to quality control using the SAMtools suite [27]. Consensus fasta sequences were generated by employing iVar (https://andersen-lab.github.io/ivar/html/index.html, accessed on 1 June 2023). Positions with fewer than five nucleotides were designated as ‘Ns.’. Phylogenetic analysis was carried out using the Nextstrain Augur pipeline [28] as follows. Sequences were aligned to DENV1 (NC_001477.1) or DENV3 (NC_001475.2) reference genomes using MAFFT [29], and a time-resolved phylogenetic tree was constructed using IQ-Tree and TreeTime under the GTR substitution model and visualized with auspice [28]. Blast [30] was used to find DENV sequences that are closely associated with the sequenced DENV samples.

## 3. Results

From September to mid-October 2022, during a major dengue outbreak in Nepal, patient samples from local Nepalese or samples from travelers returning from Nepal were diagnosed with DENV. Among the 41 non-Nepalese (travelers and expatriates) and 28 local Nepalese patients tested, 56 were likely infected in Kathmandu and were diagnosed as DENV-positive via real-time PCR. A detailed description of this group was recently published [23]. To explore the connections between the DENV identified in these patients and to delve into the possible geographic origin of the virus, the whole genome sequencing of DENV was conducted. For each DENV1/DENV3 serotype, samples from two travelers and one local Nepalese were selected. (Table 1). Samples were chosen at random from the subset of samples exhibiting sufficiently low Ct values, indicative of a high viral load, rendering them suitable for whole genome sequencing. For each DENV1/DENV3 serotype, samples from two travelers and one local Nepalese were selected (Table 1). DENV1 and DENV3 samples achieved 97 ± 2% and 99 ± 0.4% of their complete genomes, with a mean sequencing depth of approximately 1200 and 380 nucleotides, respectively. The sequences are available in Genbank (accession #OQ714403-OQ714408).

**Figure 1:**
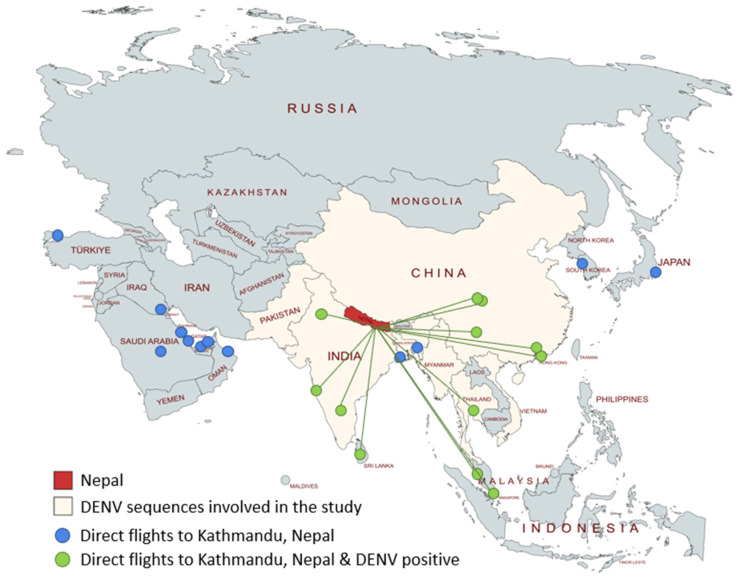
Travel patterns and availability of DENV sequences in Nepal and neighboring countries. Nepal (red) and dengue-endemic neighboring countries of Nepal (white) from which DENV1 and DENV3 sequences were publicly available. Blue circles denote countries/cities from which direct flights to Kathmandu are available. Green circles denote DENV-endemic countries/cities from which direct flights to Kathmandu were available.

Phylogenetic analyses were performed separately for the samples belonging to each DENV1/DENV3 serotype. The analyses included the sequenced Israeli travelers and local Nepalese samples (DENV1: n = 3; DENV3: n = 3), top Blast hits obtained for each sequenced sample (DENV1: n = 60; DENV3: n = 60), representative DENV1/DENV3 sequences from the neighboring countries of Nepal (Figure 1; DENV1: n = 30; DENV3: n = 31), and representative DENV1/DENV3 sequences from additional countries worldwide (DENV1: n = 44; DENV3: n = 42; https://nextstrain.org/dengue, accessed 1 June 2023). The DENV3 phylogenetic tree also included a single sample from an inflicted Israeli traveler who visited Nepal in 2019 [6].

**Figure 2:**
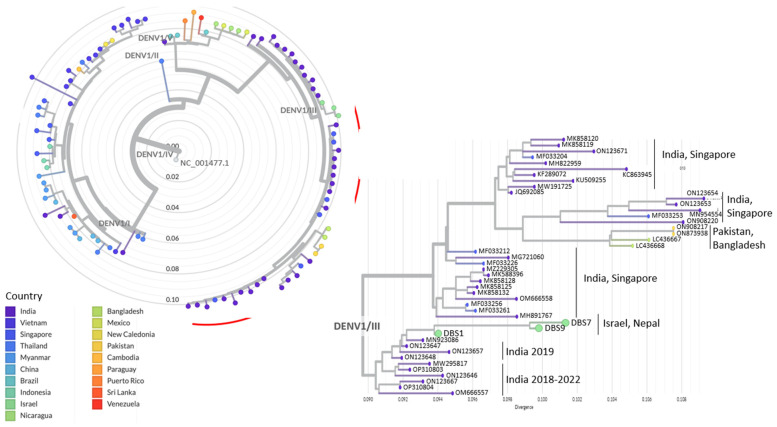
Phylogenetic analyses of DENV1 whole genome sequences.

Within the DENV1 serotype phylogenetic analysis, all three DENV1 samples from the travelers and local Nepalese sequenced in this study were clustered together under the DENV1/III clade. This clade also included sequences from India, Singapore, Pakistan, and Bangladesh (Figure 2). The samples from Israeli and Nepalese individuals sequenced in this study, exhibited the closest clustering with sequences from India between 2018 and 2022. This suggests a pronounced affinity with sequences from India in recent years.

**Figure 3:**
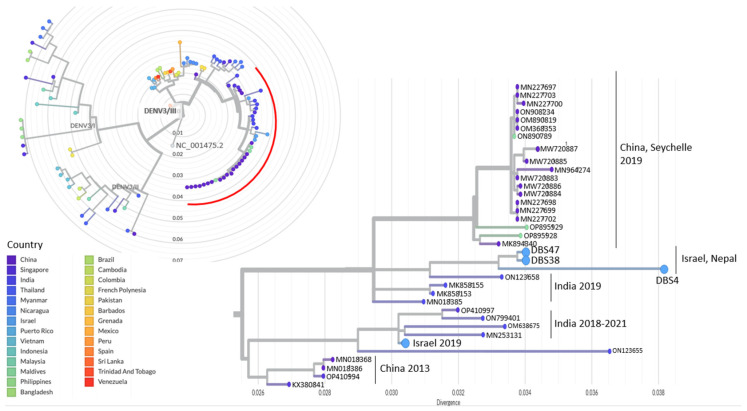
Phylogenetic analyses of DENV3 whole genome sequences.

Within the DENV3 serotype phylogenetic analysis, all three DENV3 samples from the travelers and local Nepalese sequenced in this study were clustered under the DENV3/III clade. This clade also included sequences from India, Pakistan, Singapore, China, Maldives, and Thailand (Figure 3). The samples from Israeli and Nepalese individuals sequenced in this study exhibited the closest clustering with sequences from India from India between 2016 and 2019. The additional sequence from the Israeli traveler returning from Nepal in 2019 clustered most closely with a group of sequences from India, sampled in 2018–2021, and a sequence from Singapore in 2019. 

The phylogenetic tree represents the divergence of samples sequenced in the current study (n = 3; light green) and additional global sequences (n = 103). The complete tree is presented as a radial tree, with each DENV1 genotype indicated on its respective branch (DENV1 genotype annotations sourced from https://github.com/nextstrain/dengue, accessed on 1 June 2023). The nodes in the tree are colored according to the country of origin of each sequence. The DENV1 reference used as the root of the tree is indicated (NC_001477.1). The section highlighted by a red arc in the tree represents an enlarged portion, displayed as a rectangular tree, focusing on the samples sequenced in this study and their clustered sequences. The corresponding country of origin for each cluster of sequences is indicated.

The phylogenetic tree represents the divergence of samples sequenced in the current study (n = 3; light blue) and additional global sequences (n = 103). The complete tree is presented as a radial tree, with each DENV3 genotypes genotype indicated on its respective branch (https://github.com/nextstrain/dengue, accessed 1 June 2023). The nodes in the tree are colored by the country of origin of each sequence. The DENV3 reference used as the root of the tree is indicated (NC_001475.2). The section highlighted by a red arc in the tree represents an enlarged portion displayed as a rectangular tree, focusing on the samples sequenced in this study and their clustered sequences. The corresponding country of origin for each cluster of sequences is indicated.

## 4. Discussion

During October 2022, more than 40 travelers either in or returning from Nepal, all infected in Kathmandu, were diagnosed with DENV1 and DENV3 in Israel, with a higher incidence observed for DENV1 [23]. Nepal serves as a destination for a substantial influx of travelers, exposing them to the risk of dengue infection. This increased risk not only jeopardizes the well-being of these travelers but also increases the potential for virus transmission to their respective home countries. Consequently, this phenomenon significantly augments the enduring global burden associated with dengue. Certainly, the incidence of indigenous dengue infections in Europe is increasing, with the latest documented cases primarily reported in France, Italy, and Spain in 2023 [14]. Given that Kathmandu serves as a hub for international flights, the likely pathways for the introduction of these DENV serotypes into Kathmandu could be either via land routes, predominantly from India, or via international flights. Flights to Kathmandu from dengue-endemic countries primarily originate from five main nations: India, Thailand, China, Malaysia, Sri Lanka, and Singapore (Figure 1). 

Phylogenetic analyses were conducted to investigate the origin of DENV1 and DENV3 identified in the returning travelers diagnosed in Israel and the local Nepalese samples. The analysis included additional DENV1/DENV3 genomes from Nepal’s neighboring countries—China, India, Pakistan, and Bangladesh—as well as additional globally representative samples. The analyses identified that the DENV1 sequenced samples belong to the DENV1/III clade and the DENV3 sequenced samples belong to the DENV3/III clade. In both the DENV1 and DENV3 analyses, the sequences from returning travelers were clustered with the local Nepalese samples. This implies that the travelers were most likely infected in Nepal, and that DENV1/III and DENV3/III are clades circulating in the country. Remarkably, in both DENV1 and DENV3 analyses, the samples from travelers and local Nepalese demonstrated the closest clustering with sequences originating from India. Indeed, according to the reported phylogenetic analyses of global sequences (https://nextstrain.org/dengue/denv1, accessed 1 June 2023), DENV1/III is the predominant DENV1 clade in India and the exclusive clade identified in Bangladesh and Pakistan. Conversely, in China, another sizable country bordering Nepal, and in Thailand, the DENV1/I clade is the prevailing clade, with the DENV1/III clade constituting less than a quarter of the clades. Collectively, these data indicate that the origin of DENV1 in Nepal might be linked to India, potentially through the ongoing transmission of this particular strain.

The examination of global sequences via phylogenetic analysis of DENV3 (https://nextstrain.org/dengue/denv3, accessed on 1 June 2023) reveals that the DENV3/III clade is the predominant clade in India, the sole clade identified in Pakistan, and notably absent in Bangladesh, as per reported sequences. Collectively, this implies that DENV3 may also have originated from India. Interestingly, the DENV3/III clade constitutes nearly half of the DENV3 clades identified in China. In the case of DENV3, unlike DENV1, sequences collected in China were found within the same cluster as the sequences from Israeli travelers, local Nepalese, and those from India. This observation might suggest additional transmission dynamics between India and China involving viruses belonging to this specific clade.

It should be noted that this analysis encompasses global samples that have been sequenced and are accessible in public databases. While the data were retrieved from numerous countries neighboring Nepal, there might be variations in the availability of data from different countries and some countries with missing data altogether, leading to potential disparities.

A noteworthy aspect of this study is that the analyzed samples were from patients inflicted in Kathmandu, which is located at a high elevation of about 1400 m above sea level. Due to climatic changes in the past decade dengue, vectors *Aedes aegypti* and *Aedes albopictus*, typically linked to tropical regions and lower elevations, were identified in the Kathmandu Valley. Kathmandu has emerged as an optimal breeding ground for *Aedes* mosquitoes, driven by the region’s climatic changes, urbanization, travel from dengue-endemic countries, and inadequate health infrastructure—all contributing factors to the dissemination of dengue [16]. Furthermore, dengue outbreaks typically peak at the end of the monsoon season (September–October), aligning with the peak of the popular trekking season that attracts thousands of travelers to Nepal. Although some of the Israeli travelers diagnosed in 2022 contracted the infection in Kathmandu, symptoms appeared while they were trekking in the high-elevation mountains, evident from the necessitated helicopter rescue [19,23]. This phenomenon highlights the additional risk posed by trekkers in Nepal, potentially serving as a contributing factor to the spread of the virus to higher elevations.

Top of Form

In conclusion, as the global concern for dengue transmission continues to grow, encompassing higher altitudes such as Kathmandu in Nepal, the potential spread of the virus by travelers to and from neighboring countries and beyond raises concerns for the further expansion of DENV on a global scale. This is particularly significant, given the presence of DENV vectors in new regions, including Europe, where a rising number of autochthonous transmissions have been documented in recent years. In this regard, Israel, characterized by a predominantly Mediterranean climate, similar to other South European countries, could be at increased risk for future autochthonous transmissions. Therefore, in addition to sustaining efforts for vector and virus control within Nepal, it is crucial to intensify and maintain vigilant surveillance of DENV in Nepal and neighboring countries. This surveillance, which includes travelers as sentinels, plays a highly significant role in effectively managing and controlling the spread of the virus.

## Figures and Tables

**Table 1:** DENV1 and DENV3 samples selected for sequencing. Patient information and sequencing coverage were obtained for selected samples. Ct (cycle threshold) values of real-time PCR test for DENV1/3 and sequencing coverage parameters (% coverage of the whole genome and mean depth of sequencing) are indicated per patient sample; DBS—dried blood spot.

	Name	Ct Value	Traveler/Local	% Coverage	Mean Depth
DENV1	DBS9	24.5	local	99.5	3647
	DBS1	16.08	traveler	85	62.9
	DBS7	22.82	traveler	83	48
DENV3	DBS4	22.03	local	94.1	77.2
	DBS38	23.65	traveler	99.6	565.6
	DBS47	23.3	traveler	99.5	514.7

## Data Availability

Sequences generated in this study are available in Genbank (accession #OQ714403-OQ714408).
